# Controlled saturation magnetization transfer for reproducible multivendor variable flip angle T_1_ and T_2_ mapping

**DOI:** 10.1002/mrm.28109

**Published:** 2019-12-17

**Authors:** Rui Pedro A. G. Teixeira, Radhouene Neji, Tobias C. Wood, Ana A. Baburamani, Shaihan J. Malik, Joseph V. Hajnal

**Affiliations:** ^1^ Center for the Developing Brain School of Biomedical Engineering and Imaging Sciences King’s College London London United Kingdom; ^2^ Biomedical Engineering Department School of Biomedical Engineering and Imaging Sciences King’s College London London United Kingdom; ^3^ Magnetic Resonance Research Collaborations Siemens Healthcare Limited Frimley United Kingdom; ^4^ Department of Neuroimaging King’s College London London United Kingdom

**Keywords:** CSMT, DESPOT, JSR, magnetization transfer, relaxometry, reproducibility, T_1_ mapping, T_2_ mapping

## Abstract

**Purpose:**

The widespread clinical application of quantitative MRI has been hindered by a lack of reproducibility across sites and vendors. Previous work has attributed this to incorrect B_1_ mapping or insufficient spoiling conditions. We recently proposed the controlled saturation magnetization transfer (CSMT) framework and hypothesized that the lack of reproducibility can also be attributed to magnetization transfer effects. This work seeks to validate this hypothesis and demonstrate that reproducible multivendor single‐pool relaxometry can be achieved with the CSMT approach.

**Methods:**

Three healthy volunteers were scanned on scanners from 3 vendors (GE Healthcare, Philips, Siemens). An extensive set of images necessary for joint T_1_ and T_2_ estimation were acquired with (1) each vendor default RF pulses and spoiling conditions; (2) harmonized RF spoiling; and (3) harmonized RF spoiling and CSMT pulses. Different subsets of images were used to generate 6 different T_1_ and T_2_ maps for each subject’s data from each vendor. Cross‐protocol, cross‐vendor, and test/retest variability were estimated.

**Results:**

Harmonized RF spoiling conditions are insufficient to ensure good cross‐vendor reproducibility. Controlled saturation magnetization transfer allows cross‐protocol variability to be reduced from 18.3% to 4.0%. Whole‐brain variability using the same protocol was reduced from a maximum of 19% to 4.5% across sites. Both CSMT and native vendor RF conditions have a reported variability of less than 5% for repeat measures on the same vendor.

**Conclusion:**

Magnetization transfer effects are a major contributor to intersite/intrasite variability of T_1_ and T_2_ estimation. Controlled saturation magnetization transfer stabilizes these effects, paving the way for the use of single‐pool T_1_ and T_2_ as a reliable source for clinical diagnosis across sites.

## 1 INTRODUCTION

Magnetic resonance imaging has established itself as one of the main workhorses of neuroimaging due to its ability to generate high, soft‐tissue contrast that is sensitive to different aspects of tissue microstructure. With its widespread adoption, researchers have quickly recognized the advantages of pooling resources to increase statistical power of their studies.^1^, ^2^ However, there is still some controversy about the level of intersite comparability achievable in conventional MRI scans and how this might bias morphometric analysis.^2^, ^3^ Quantitative MRI (qMRI) seeks to tackle this issue by providing absolute measures of tissue properties, to allow measurements to be comparable across scanners and time‐points.^2^, ^4^, ^5^ However, recent work by Bojorquez et al,^6^ which collated the range of normative spin‐lattice recovery (T_1_) and spin‐spin (T_2_) relaxation times for brain reported throughout the literature at 3 T, found an extremely wide range of values for similar tissues. As an example, white matter (WM) T_1_ values ranged from 699 ms to 1735 ms,^6^ which clearly undermines the promise of qMRI as a tool to obtain comparable and reproducible measures. In previous work, Stikov et al^7^ also demonstrated systematic differences between Look‐Locker, variable flip angle (VFA,) and inversion‐recovery T_1_ mapping approaches in vivo. In their work, they concluded that these discrepancies are due to both incomplete spoiling and inaccurate RF field (B_1_) mapping, and proposed calibrating relaxometry protocols against an inversion‐recovery reference method. More recently, Lee et al^4^ sought to establish intravendor and intervendor reproducibility of T_1_ times at 3 T of a specific VFA protocol (multiparametric mapping),^2^ which is used widely and is extensively optimized.^4^ In their study, they identified a systematic bias of 7.8%‐10.0% between the 3T Philips Achieva (Best, Netherlands) and the 3T Siemens MAGNETOM Trio (Erlangen, Germany) scanners. In our own work,^8^ we suggested that discrepancies of single‐pool T_1_ measures across the literature might be due to magnetization transfer (MT) processes that intrinsically occur in brain tissues.^9^ Unlike single‐pool models, which assume an unique source of magnetization inside each voxel, an MT system is typically characterized by a pool of mobile protons (e.g., liquid water) in close contact with a proton‐rich matrix (restricted pool[s] of protons),^9^, ^10^, ^11^ allowing exchange between both pools but where the T_2_ of the restricted pool is so short that its signal decays before it can be measured. With this in mind, we highlighted that VFA relaxometry methods acquire data using different RF pulse power in each component acquisition, and this results in variable and generally uncontrolled partial saturation conditions for the bound pool(s).^8^ To address this issue, the controlled saturation magnetization transfer (CSMT) approach was proposed. It uses nonselective RF pulses tailored to equalize saturation power across all measurements. In other words, by allowing a 5º flip angle (FA) image to be acquired with the same RF power as a 60º FA image, we are able to stabilize MT effects in a VFA experiment.^8^ In this work, we sought to validate our hypothesis that the lack of T_1_ and T_2_ mapping reproducibility across studies can be attributed to MT effects. Focusing on VFA, we implemented the CSMT framework on 3 different MRI scanners from 3 different vendors (GE Healthcare [Milwaukee, Wisconsin], Philips [Best, Netherlands], and Siemens [Erlangen, Germany]) and performed a traveling head study to explore: (1) systematic differences in obtained T_1_ and T_2_ values that depend on both the vendors used and the particular protocol used; (2) the contribution of harmonizing RF spoiling conditions on these discrepancies; and (3) the potential of harmonizing MT saturation effects through CSMT to significantly increase reproducibility of the estimated parameters across both vendor and protocol.

## 2 METHODS

We sought to establish the stability of VFA T_1_ and T_2_ estimation procedure using both native and CSMT RF pulse types, and to establish the effect of RF spoiling, as this is typically associated as the cause of discrepancy among qMRI methods. Three different levels of reproducibility were tested:

1. Reproducibility across protocols: As we have discussed in previous work,^8^, ^12^ when the measured sample is well‐characterized by a single‐pool model, the use of different FAs is expected to change the variance of estimation but not the average estimated T_1_ and T_2_.
2. Reproducibility across vendors: Here we evaluated, for a given protocol, how reproducible they are across different vendors. As lack of reproducibility in qMRI has previously been attributed to differences in RF spoiling,^7^, ^13^ we also evaluated the effect of harmonizing RF‐spoiling conditions.
3. Reproducibility across repeated measures: We sought to also establish test/retest reproducibility of repeated estimation of T_1_ and T_2_ on the same vendor as well as across different vendors.

To assess all 3 points, we made use of a variability metric between 2 measures (*m_i_* and *m_j_*), defined as their percentage difference normalized by their mean (${\text{variability}}_{i,j}=100\times \left(m_{i}-m_{j}\right)/0.5\left(m_{i}+m_{j}\right)$. To compare several measures (e.g., different FA protocols and/or vendors) simultaneously, we used a deviation metric, defined here as the percentage difference between a single measure relative to the mean of all measures (${\text{deviation}}_{i}=100\times \left(m_{i}-\bar{m}\right)/\bar{m}$, where $\bar{m}=\frac{1}{N}\sum m_{i}$ is the mean of all measures).

Three male healthy and experienced volunteers (mean age 25 years, range 23‐28 years) were scanned on 3 3T MRI systems: a GE Discovery MR750 (GE Healthcare) located at King’s College Hospital (London, United Kingdom), and a Philips Achieva (Philips Healthcare) and a Siemens Biograph‐mMR, both located at St. Thomas’ Hospital (London, United Kingdom). The scanners are located in different departments of the same institution and will be referred to, throughout the text, as vendor A (Philips), vendor B (Siemens), and vendor C (GE). This allows us to both be more succinct in the description of the different vendors, and emphasize that MT effects in VFA qMRI are not a vendor‐specific issue. All scanning was obtained after written informed consent according to the local ethics guidelines of each site. In all scanners, data were acquired at 1‐mm^3^ isotropic resolution with parallel imaging acceleration factor of 2 (SENSE or GRAPPA depending on the vendor). Different receive array coils were used for different scanners, the data of vendors A and C were acquired with 32 element arrays, whereas vendor B used an array of 12 elements. The TR/TE values were fixed at TR/TE = 7.0/3.5 ms for all VFA images. Flip angles of 3º, 7º, 11º, and 15º were obtained for spoiled gradient‐recalled images (SPGR, also known as T_1_‐FFE or FLASH). Balanced SSFP (bSSFP, also known as balanced‐FFE or TrueFISP) data were obtained at FAs of 5º, 25º, and 45º with RF‐phase increments of 180º between consecutive pulses. Balanced SSFP is known to have a strong dependence on static (B_0_) field inhomogeneities, giving rise to characteristic “black‐band” profile.^14^, ^15^ To address this, an extra 45º FA with 0º RF increment (which effectively shifts the banding profile) was also acquired and has been previously shown to allow B_0_ field estimation.^12^, ^16^ Transmit field inhomogeneities were measured using each vendor’s default method (Bloch‐Siegert shift,^17^ actual flip‐angle imaging,^18^ and saturation prepared turbo field echo^19^, ^20^). All images were measured twice: with the default RF‐pulse for each vendor and with a nonselective 3‐band CSMT pulse (achieved by modulating a 2.5‐ms Gaussian‐shaped pulse) designed for a target RMS B_1_ of 1.6 uT^8^ with ±6 kHz off‐resonance saturation bands. It is known^12^, ^13^, ^21^ that sufficient RF spoiling is one of the leading parameters that could hinder the reproducibility of T_1_ estimation when SPGR images are used. To avoid this as a confounding factor, the software of the different scanners was modified to allow a quadratic phase increment of 50º between RF pulses, which was chosen due to its demonstrated stability to imperfections.^13^ To highlight the effect of RF spoiling on the overall reproducibility, in 1 volunteer, SPGR images were also obtained with each of the default vendor settings (quadratic RF increments of 150º, 50º, and 115º). That same volunteer was also scanned twice at each scanner to assess the test/retest variability. No care was taken in order to unify gradient spoiling moments across all vendors.

Estimation of T_1_ and T_2_ values from the collected data was obtained using the joint system relaxometry (JSR) approach.^12^ As with conventional DESPOT,^22^ JSR makes use of SPGR and balanced SSFP images; however, both signal models are evaluated simultaneously, boosting estimation precision compared with the conventional 2‐step approach.^12^ Parameter maps were estimated using all measures as well as 5 other different subsets of the measured FAs (Table 1), emulating the effect of acquiring different qMRI protocols. Joint system relaxometry is not an available commercial package from any of the manufacturers, and all data were processed on in‐house developed software written in MATLAB 2017b (MathWorks, Natick, Massachusetts).Set Table 1 as one or two column in PDF

**Table 1:** Spoiled gradient‐recalled echo and balanced SSFP flip‐angle volumes acquired

	SPGR (°)				bSSFP (°) ‐ 180°			bSSFP (°) ‐ 0°
All FA	3	7	11	15	5	25	45	45
Subset 1	3	7	11	15	5	25	45	45
Subset 2	3	7	11	15	5	25	45	45
Subset 3	3	7	11	15	5	25	45	45
Subset 4	3	7	11	15	5	25	45	45
Subset 5	3	7	11	15	5	25	45	45

Note

The subsets used for joint T_1_ and T_2_ estimation are not new data but extracted from the all‐measures superset.

Abbreviations: bSSFP, balanced SSFP; FA, flip angle; SPGR, spoiled gradient‐recalled echo.

All subjects were analyzed completely independently. First, all images for a single subject were aligned to a common space (rigid transformation) using FSL‐FLIRT.^23^, ^24^ Normalized mutual information was used as the cost function to align the different contrasts. After alignment, for each volunteer, the 15º SPGR measurement (acquired with CSMT conditions) from vendor A was used to generate a WM‐specific mask extracted using the FSL‐FAST^25^ algorithm. The generated mask was eroded using a 2‐mm‐radius sphere to mitigate partial volume contributions. The same mask was used to extract T_1_ and T_2_ WM‐specific distributions (histograms) from all of the computed maps. The median value of each distribution was then used to assess the variability between estimations. Median was chosen, as some WM distributions are skewed and the median was found to be a better indicative metric of the distribution peak. When comparing several distributions, we also defined worst‐case variability as the biggest observed difference between the extracted medians. For analysis of the test‐retest data, we used the variability and deviation measures calculated voxel‐wise for all voxels in the WM mask for each subject.

## 3 RESULTS

**Figure 1:**
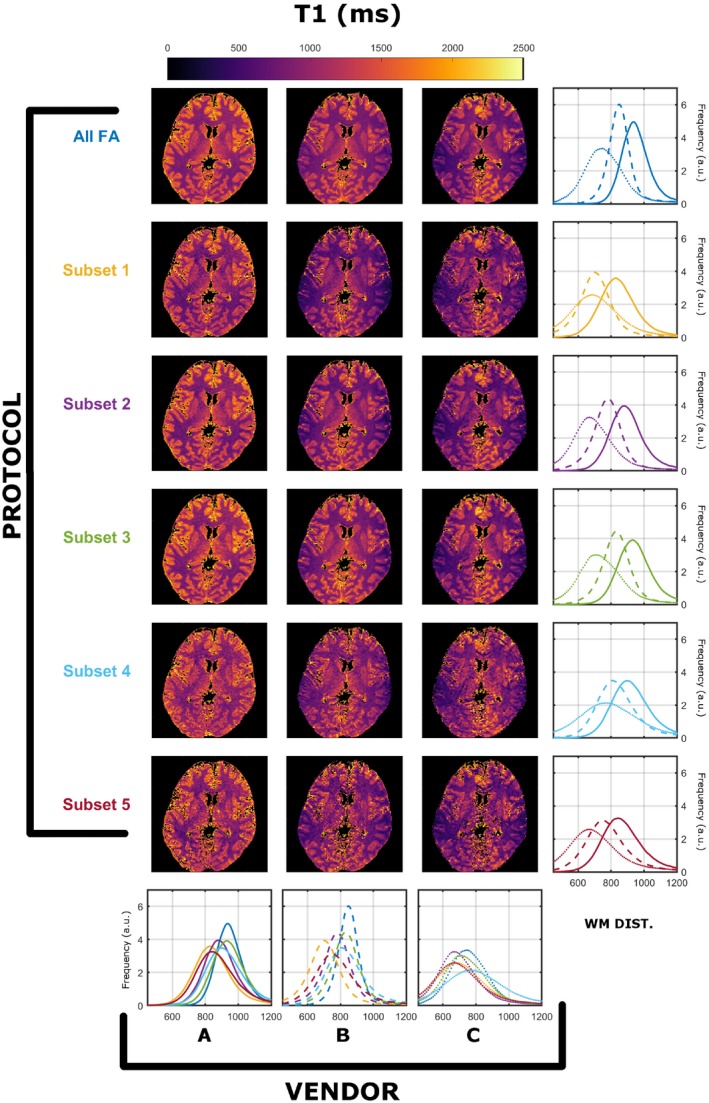
Comparison of T_1_ (in milliseconds) compared across vendors using the data acquired from native RF spoiling and saturation conditions. All histograms were obtained from a single white matter (WM) mask extracted as described in the Methods. Each color represents a different protocol, as per Table 1. Solid, dashed, and dotted lines correspond to vendor A, B, and C WM‐specific distributions, respectively

**Figure 2:**
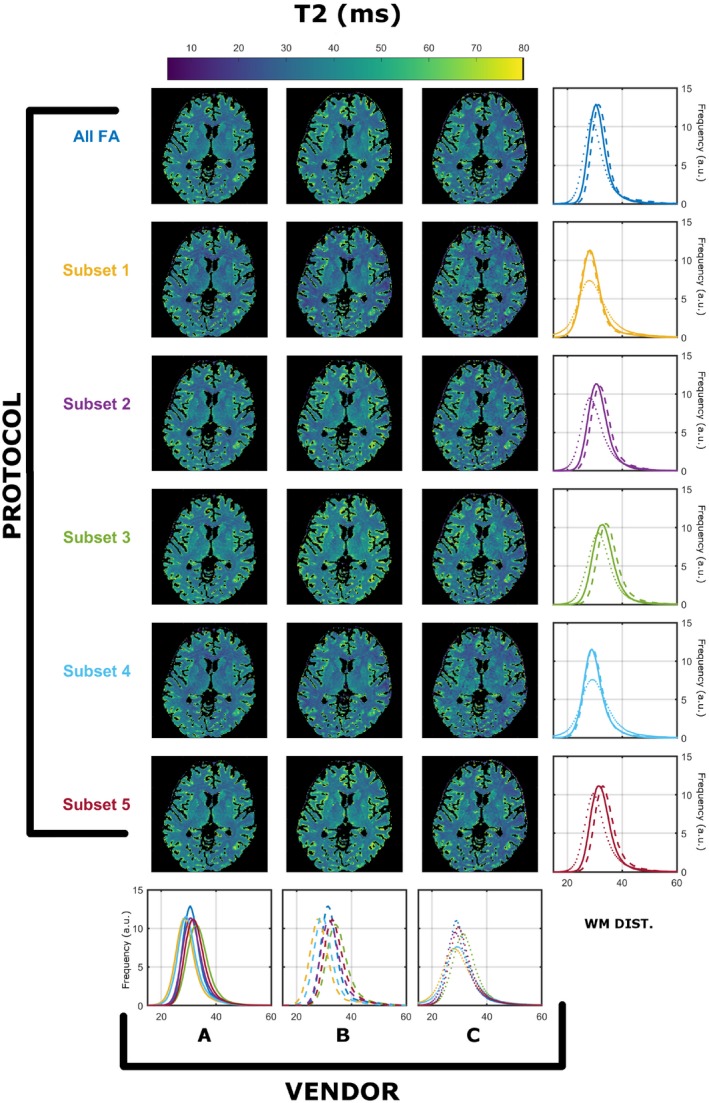
Comparison of T_2_ (in milliseconds) compared across vendors using the data acquired from native RF spoiling and saturation conditions. All histograms were obtained from a single WM mask extracted as described in the Methods. Each color represents a different protocol, as per Table 1. Solid, dashed, and dotted lines correspond to vendor A, B, and C WM‐specific distributions, respectively

Similar findings were found for all 3 subjects. The registration process resulted in maximal corrections of 21 mm in translation and 28º in rotation. The WM masks for the 3 subjects contained 84 960, 109 378, and 111 990 voxels, respectively. Results from a single subject (subject 2) was used for detailed exploration, as all subjects demonstrated similar results, and a summary for all subjects is presented in Figure 9. Figures 1 and 2 summarize the T_1_ and T_2_ maps, respectively, obtained using the preferred sampling conditions of each MRI system (each scanner has its own spoiling regime and RF pulse). In both Figures, each column corresponds to a specific vendor and each row to a specific protocol, as per Table 1. The histograms in the rightmost column overlay WM‐specific distributions estimated from each protocol as obtained from each vendor. The histograms in the bottom row highlight a direct comparison among WM distributions obtained for each vendor for all protocols. On every histogram, vendors are designated by line style (A, solid; B, dashed; C, dotted), whereas different protocols are represented by different colors, as per Table 1. Throughout this work we focused on WM‐specific distributions in order to avoid registration errors and partial volume effects as confounders.

Figure 1 shows that there are systematically different estimated T_1_ WM distributions that depend on both the vendor and the subset used. Focusing on the medians of the distirbutions, the observed worst‐case variability among protocols is 11.6% for A, 18.3% for B, and 13.6% for C. Although the maximum variability among vendors is 25.6% (subset 2), and the minimum is 14.7% (subset 5). Figure 2 indicates that similar observations can be made for estimated T_2_ ditributions, where the observed worse‐case variability among protocols is 14.2% for A, 20.0% for B, and 9.0% for C, and among scanners is a maximum of 10.0% (subset 5) and a minimum of 2.0% (subset 1). Furthermore, the average T_2_ values are systematically lower then expected from the T_2_ values reported in previous studies.^26^

**Figure 3:**
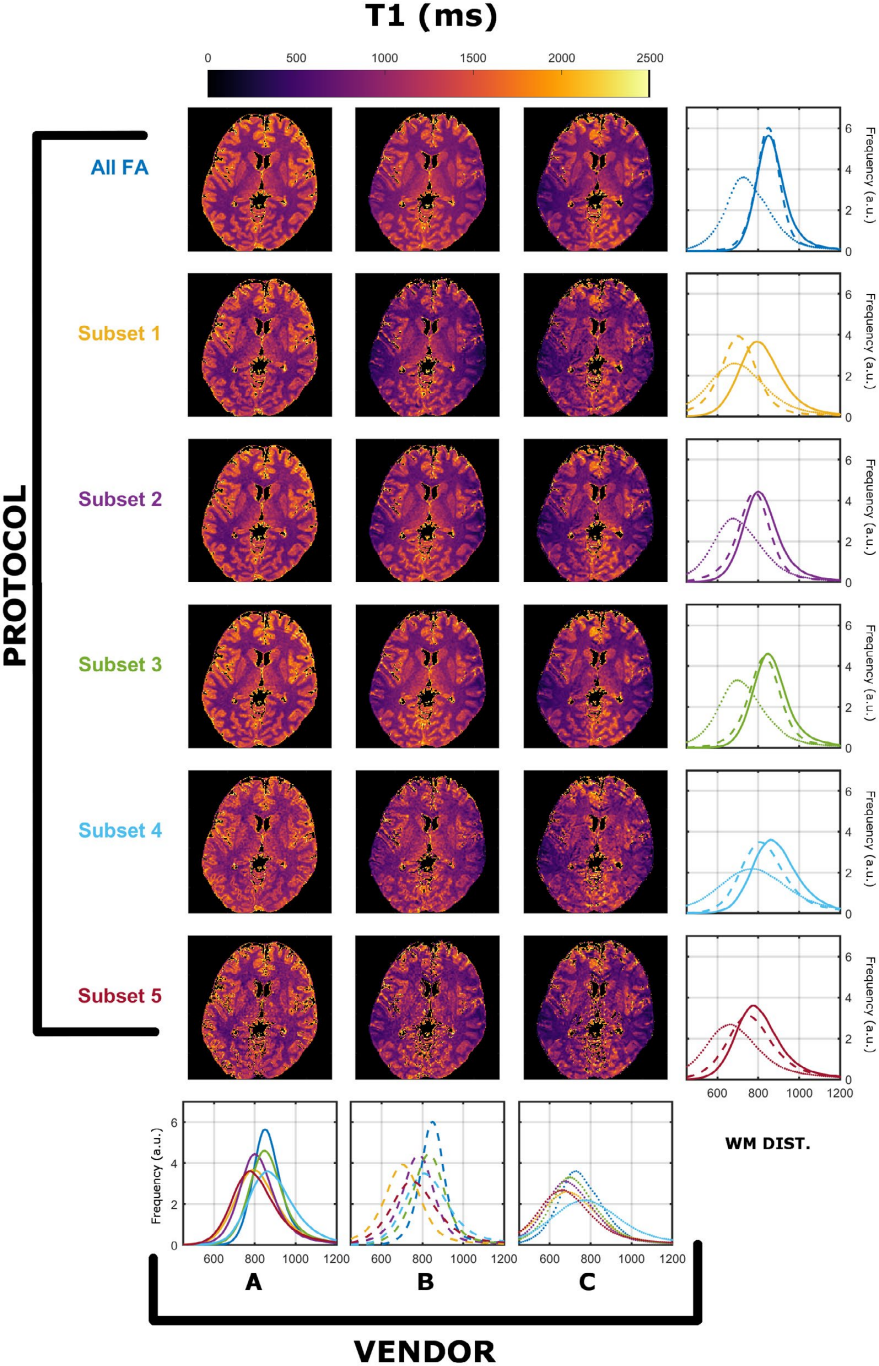
Cross‐vendor T_1_ (in milliseconds) estimation comparison of the data acquired from each scanner’s native saturation conditions and harmonized RF spoiling of 50º. All histograms were obtained from a single WM mask extracted as described in the Methods. Each color represents different protocols, as per Table 1. Solid, dashed, and dotted lines correspond to vendor A, B, and C WM‐specific distributions, respectively

**Figure 4:**
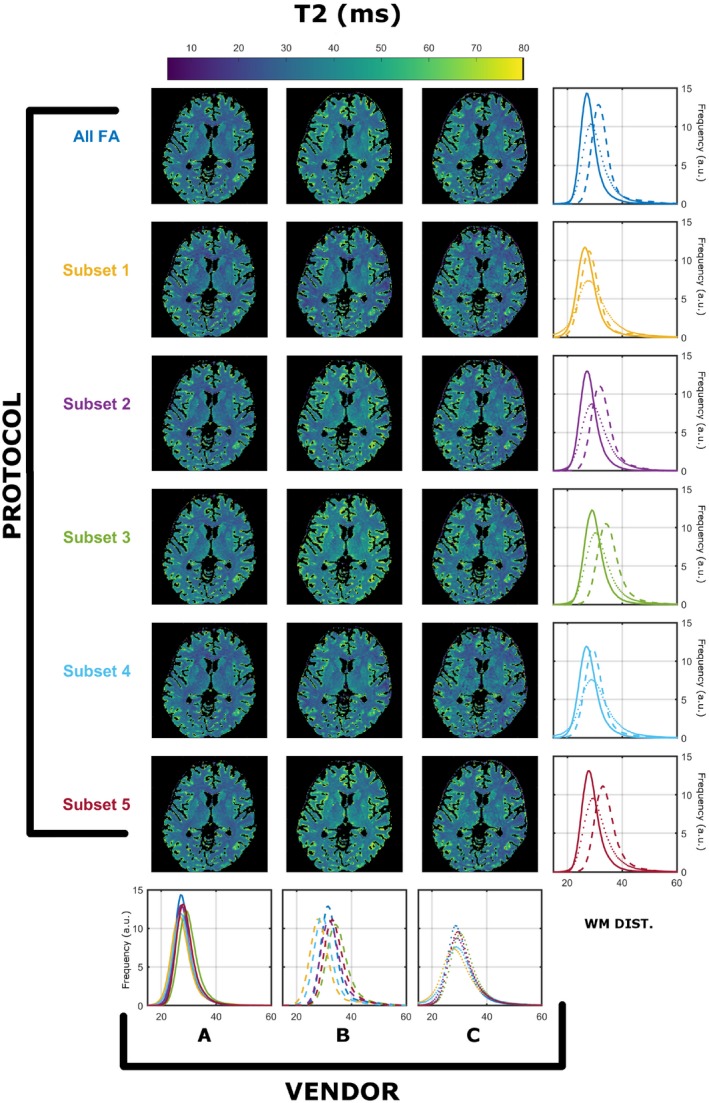
Cross‐vendor T_2_ (in milliseconds) estimation comparison of the data acquired from each scanner’s native saturation conditions and harmonized RF spoiling of 50º. All histograms were obtained from a single WM mask extracted as described in the Methods. Each color represents different protocols, as per Table 1. Solid, dashed, and dotted lines correspond to vendor A, B, and C WM‐specific distributions, respectively

Figures 3 and 4 demonstrate the same summary of results as presented for Figures 1 and 2; however, data were acquired with harmonized RF spoiling of 50º in all systems. Regarding T_1_ estimation with unified RF spoiling, there is a much greater agreement between vendors A and B. However, the observed worse‐case variability among protocols is 9.5% for A, 18.3% for B, and 12.9% for C. The maximum variability among scanners is 17.5% (subset 3), and the minimum is 11.4% (subset 4). Figure 4 shows that similar observations can be made for estimated T_2_ distributions, where the observed worse‐case variability among protocols is 8.8% for A, 20.0% for B, and 7.5% for C, whereas the maximum variablity among vendors ranges between 16.8% (subset 5) and 6.0% (subset 1). This result is interesting, as it seems to imply that harmonizing RF spoiling increases the cross‐scanner reproducibility of T_2_; however, care must be taken when analyzing these results. Because the JSR estimation is a joint T_1_ and T_2_ estimation approach, we speculate that this is a just a result of the particular interaction of the RF spoiling conditions and the MT effects that have not been controlled. As in Figure 2, median WM T_2_ values are lower than expected from the T_2_ values reported in previous studies.^26^ The differences in variability among different vendors are expected, as the RF pulses used have different MT properties. For example, vendor B used a short nonselective RF pulse (0.1 ms), and hence will suffer most from MT effects. This varibility, therefore, should not be used as a metric of vendor performance, as different pulse choices/sequences are available that would affect the number reported in this study.

**Figure 5:**
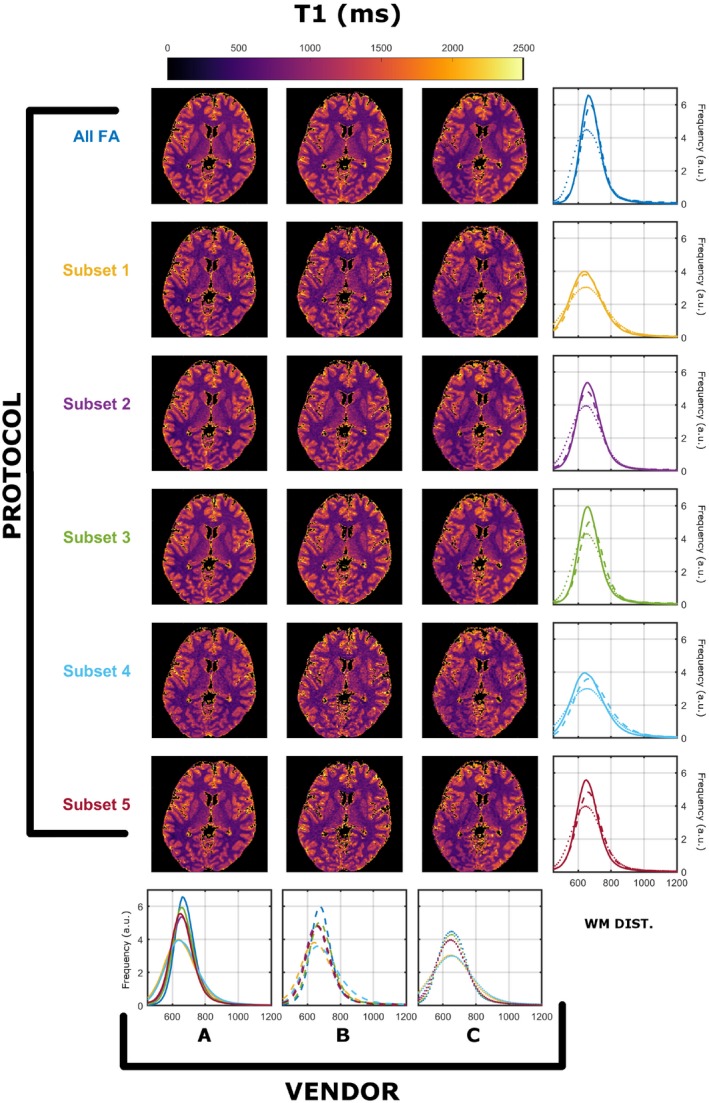
Cross‐vendor T_1_ (in milliseconds) comparison of the data acquired from both harmonized RF spoiling and controlled saturation magnetization transfer (CSMT) conditions. All histograms were obtained from a single WM mask extracted as described in the Methods. Each color represents different protocols, as per Table 1. Solid, dashed, and dotted lines correspond to vendor A, B, and C WM‐specific distributions, respectively

**Figure 6:**
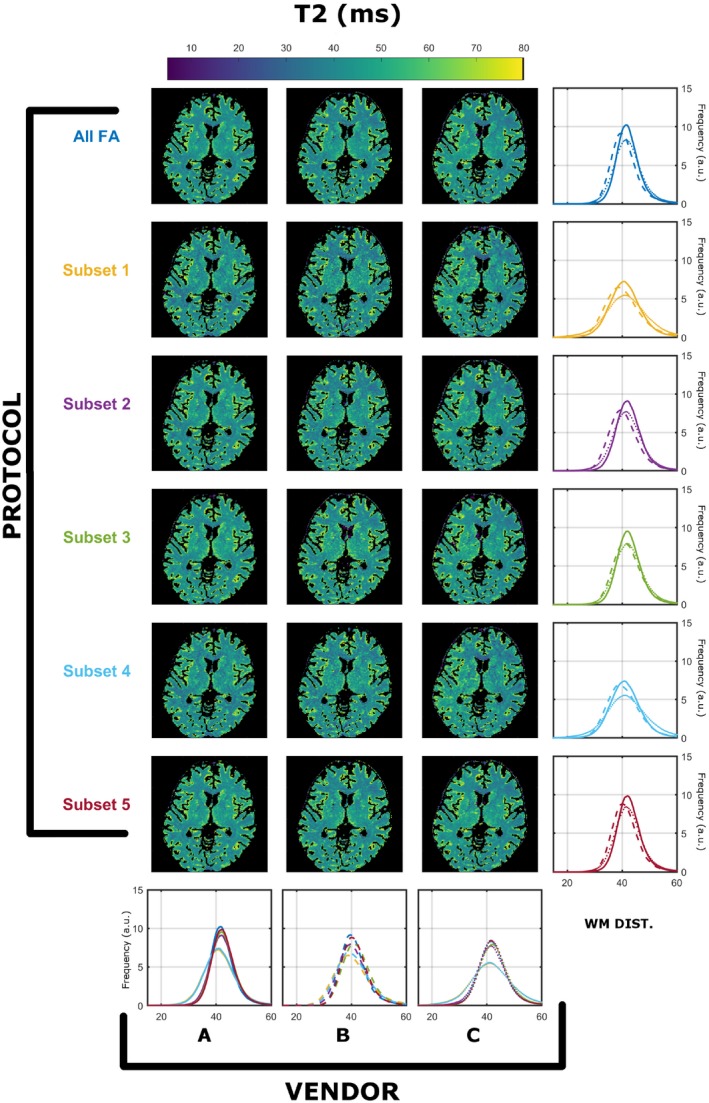
Cross‐vendor T_2_ (in milliseconds) comparison of the data acquired from both harmonized RF spoiling and CSMT conditions. All histograms were obtained from a single WM mask extracted as described in the Methods. Each color represents different protocols, as per Table 1. Solid, dashed, and dotted lines correspond to vendor A, B, and C WM‐specific distributions, respectively

Figures 5 and 6 demonstrate the same summary of results for data acquired with harmonized RF spoiling and RF saturation with CSMT conditions. The T_1_ estimation has, under such conditions, much greater agreement among all 3 vendors. The observed worst‐case variability among protocols is 4.0% for A, 3.5% for B, and 1.6% for C. The maximum variablity among scanners is 4.2% (subset 4), and the minimum is 2.0% (subsets 2 and 5). Figure 6 indicates that similar observations can be made for estimated T_2_ distributions, where the observed worse‐case variability among protocols is 3.3% for A, 4.3% for B, and 2.1% for C, whereas the maximum variablity among vendors is 4.7% for subset 2 and the minimum is 1.8% for subset 3. Median T_2_ values are now more in line with previous studies.^26^

**Figure 7:**
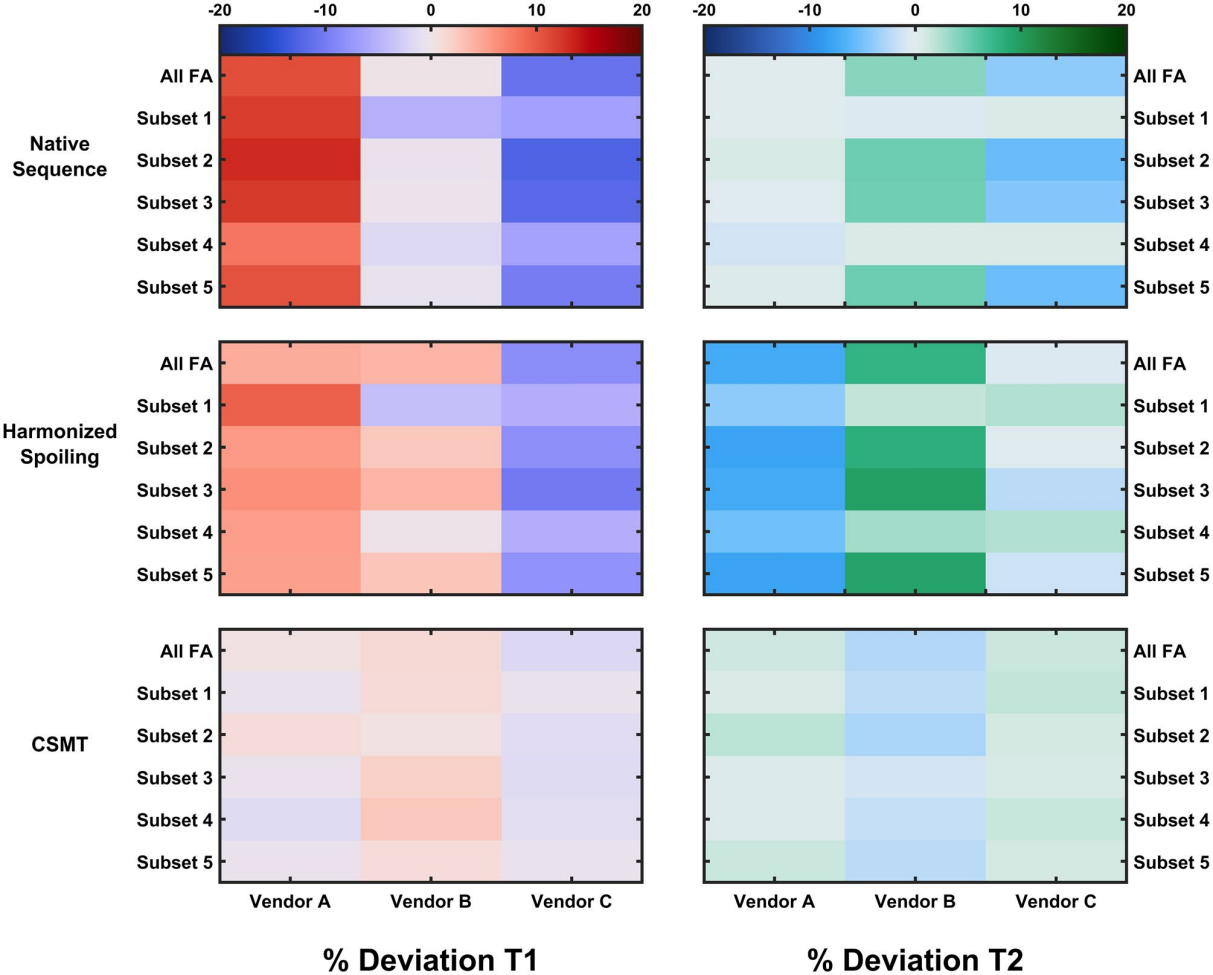
Percentage deviation of each vendor (column) and protocol (row) relative to the mean of all sites and subsets under native sequence (native RF spoiling and RF saturation sampling conditions of each site), harmonized spoiling (harmonized RF spoiling and default RF saturation of each site), and CSMT (harmonized RF spoiling and saturation using CSMT sampling). The range of reported deviations is greatly reduced when sampling under CSMT conditions

Figure 7 demonstrates the percentage deviation of the medians of the WM distributions of each vendor (columns) and protocols used (rows) relative to the mean of the distribution medians of all vendors and protocols under native sequence (native sampling conditions of each site), harmonized spoiling (harmonized RF spoiling of SPGR images), and CSMT (harmonized RF spoiling and CSMT conditions). This figure highlights that harmonizing RF spoiling reduces the deviations across vendors; however, only under CSMT conditions are the total deviations reduced to less than ±4% across protocols and vendors for both T_1_ and T_2_.

**Figure 8:**
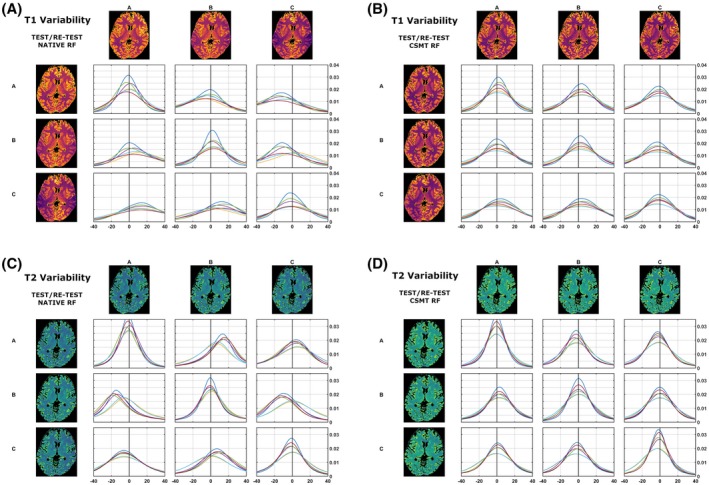
Test/retest comparison of whole‐brain variability distributions of T_1_ (A,B) and T_2_ (C,D) under harmonized RF spoiling, and native RF saturation (A,C) and CSMT RF saturation (B,D). Distributions in diagonal entries correspond to the measurement in the same scanner. Off‐diagonal histograms correspond to cross‐vendor variability. Variability is defined in the Methods as 100‐times difference/mean of each pairwise comparison. Histogram colors correspond to the different subsets used, as per Table 1

Figure 8 presents the results for test/retest variability of the estimation procedure under harmonized RF spoiling without (Figure 8A,C) and with (Figure 8 B,D) CSMT conditions. Here we summarize distributions of the whole voxel‐wise variability of the same subject for test/retest. Diagonal elements of Figure 8A,C demonstrate a variability distribution centered on zero, demonstrating good test/retest variability. However, the same is not true for a comparison among different vendors (off diagonal), and systematic nonzero centered distributions (up to 18% T_1_ and 19% T_2_) can be seen among scanners, depending on the protocol used. Under CSMT conditions (Figure 8B,D), variability distributions are centered around zero (±4% for both T_1_ and T_2_), both between repeats of the same vendor (diagonal) or among different vendors (off‐diagonal histograms), independent of the protocol used, thus highlighting the increased reproducibility allowed by the CSMT framework.

**Figure 9:**
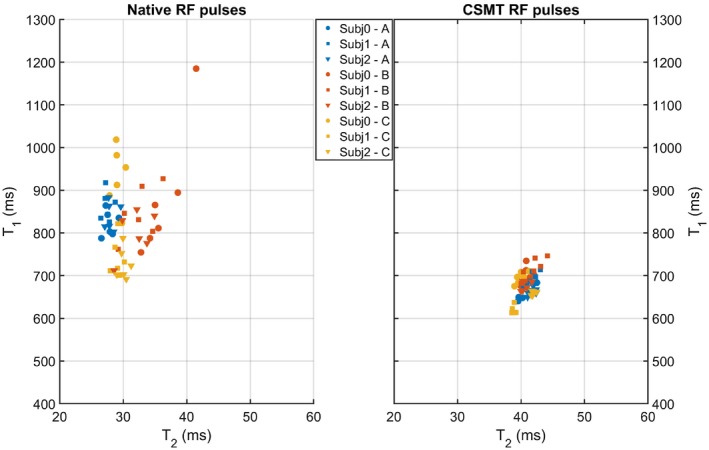
Comparison of median WM T_1_/T_2_ values for different volunteers across permutations and vendors. Each volunteer is represented as a different color. Vendors A, B, and C are represented as circles, squares, and triangles, respectively. Different points of the same volunteer/vendors represent different permutations of measurements, as per Table 1. With the CSMT RF pulses (right), the spread of T_1_/T_2_ pairs is more concentrated, and no clear difference can be observed among different sites when compared with the sampling data of native RF pulses (left)

We further validated the effect of CSMT in cross‐vendor variability on all 3 different volunteers. The results are highlighted in Figure 9, where pairs of median T_1_ and T_2_ values of the WM distributions are plotted for all 3 subjects. The 3 different subjects are represented, respectively, as circles, squares, and triangles. Different vendors are represented as different colors (A, blue; B, orange; C, yellow). Different points for the same subject/vendor combination correspond to different protocols, as per Table 1. All data presented in Figure 9 were acquired with harmonized quadratic RF phase increments of 50º. A clear difference in the spread of reported T_1_/T_2_ pairs can be found between sampling data with CSMT conditions (right) and using native RF pulses (left), demonstrating the decrease in variability when controlling for MT effects.

## 4 DISCUSSION

This work demonstrates that MT effects have a significant effect on cross‐vendor reproducibility of VFA T_1_ and T_2_ mapping. We began by demonstrating that the native sampling conditions (RF spoiling and saturation) of all 3 vendors results in variable T_1_ and T_2_ estimation, which is not consistent both for a single vendor using different protocols (i.e., different FAs) or across vendors. Harmonizing RF spoiling of SPGR images reduces variability across vendors but does not improve intravendor variability when different protocols are used to estimate the qMRI parameters. With the use of CSMT to ensure constant saturation power regardless of FA, the consistency of fitted T_1_ and T_2_ values is greatly improved, as there is reduced variability among vendors as well as for different protocols. This corroborates our hypothesis that MT saturation has a significant effect on the consistency of relaxometry measurements using VFA methods if left uncontrolled. Once MT‐induced variability is removed, results become more stable across MR systems and protocols, and therefore single‐pool qMRI (in which the signal inside each voxel is explain by a single source of magnetization) as a reproducible measurement tool becomes feasible.

Detailed quantitative comparisons were all made using a subject‐specific single WM region of interest that was produced automatically but eroded to avoid sampling close to tissue boundaries where partial volume effects might introduce variability. For the more interested reader, gray matter–specific analysis mirroring Figures 1, 2, 3, 4, 5, 6 can be found in Supporting Information Figures S1‐S6. All images, and hence parameter maps, were aligned into 1 space for each subject, to ensure that incidental effects such as minor changes in subject pose would not introduce uncontrolled variability. These pose corrections were very small (typically < 5 mm) but were found to be important in ensuring unbiased comparisons between sessions and across individuals.

Cross‐protocol variability was examined by first obtaining a superset of FA measures, then using subsets of these to generate different estimation protocols (Table 1), which should obtain the same result with varying precision.^8^, ^12^ This is different from typical reproducibility studies,^4^, ^5^, ^27^ which rely on using a single, optimized set of measurements across different sites, not exploring whether the methods are robust to different sequence parameters. The reader should not associate the reported intravendor variability as a feature of manufacturer reproducibility capability. Even within the same vendor, there are multiple variants of SPGR and balanced SSFP sequences tailored for different applications, as their software typically performs different compromises depending on the target anatomical region (e.g., brain, cardiac imaging); hence, results may vary depending on which sequence was selected to set up the qMRI protocol.

It is well‐known that RF spoiling is a critical parameter in ensuring reproducible T_1_ estimates using VFA methodologies.^7^, ^13^ The data presented in Figures 1 and 2 show T_1_ and T_2_ measurements obtained from the same human subject imaged on scanners from 3 different vendors, using a range of different protocols, and using the default RF spoiling and RF pulse shapes for each scanner. The resulting T_1_ and T_2_ maps as well as their WM distributions show systematic differences that depend on both the specific FA measurements and the vendor. We note that it is expected that different subsets have varying estimation precisions (resulting in variable distribution widths), due to their sensitivity to specific T_1_/T_2_ pairs. However, as shown from our previous work,^8^ under valid single‐pool assumptions the peaks of the distributions should not be affected by the specific subset used, but only their widths.

The estimated median T_2_ values in WM are systematically lower than what is expected for WM using spin‐echo measurements (Figure 2).^26^ These differences might then be attributed to discrepancies in RF spoiling, although Figure 4 shows the same comparison with equalized RF spoiling phase increment (50º), but with RF pulse shapes unchanged. In this case, there is still significant variability between intrascanner and interscanner measurements, which we highlight in the leftmost 2 columns of Figure 7 by computing the deviation across vendors and protocols. By comparing Figures 1, 3, and 7, we note greater agreement between vendor A and B T_1_ values when compared with C. Looking more closely at the protocols used, we designed our experiment such that all pulses have a fixed duration and varying amplitude. Vendors A and B use hard‐pulse excitations with respective durations of 0.3 ms (A) and 0.1 ms (B), whereas vendor C uses custom 1.6‐ms Shinar‐Le Roux pulses. Hence, the differences seen are consistent with the hypothesis that the differences are driven by MT effects related to the different RF saturation properties of these pulses. This emphasizes how accounting for spoiling of SPGR signals is a necessary but not sufficient condition to increase reproducibility of relaxometry studies.

Figures 5 and 6 and the bottom row of Figure 7 present the same comparison using harmonized RF spoiling and CSMT RF pulses, which equalize the RF power regardless of the FA requested. In this case, we observed an improvement in agreement among acquisition protocols and among vendors. Furthermore, the median T_2_ values in WM are now more in line with previously reported spin‐echo measurements,^26^ which is expected from numerical and experimental validation performed in our previous work.^8^ This occurs due to the fact that we are performing a simultaneous estimation of T_1_ and T_2_ parameters; therefore, any inconsistencies among the data (which are known, to a certain degree, to follow an MT model) and the assumed model (single‐pool) can be accommodated as either T_1_ and T_2_ biases. After we force the data to follow a single‐pool model, using our CSMT framework, no more systematic shifts are observed, as the assumed model correctly represents the data.

Figures 3, 4, 5, 6, 7 confirm our hypothesis that MT effects are a significant issue in multivendor studies, and that CSMT effectively allows more reproducible cross‐vendor T_1_ and T_2_ mapping studies. Although not shown, in the preliminary data obtained to set up this study, we observed that ensuring CSMT without harmonizing RF spoiling does diminish the variability among vendors, but systematic biases persist. We also note that harmonizing RF spoiling does not necessarily remove biases from imperfect spoiling; rather, it makes these effects uniform across protocols/vendors. A different approach would be to perform a polynomial correction as proposed in Preibisch and Deichmann^13^; however this would require correction parameters to be estimated for both apparent T_1_ and T_2_ for each subset of FAs and RF increment used. Another promising approach would be to use more efficient spoiling regimes, such as the “hexagonal” scheme presented by Hess et al.^28^

We further sought to identify the test/retest variability of harmonized RF spoiling conditions as well as CSMT conditions for both T_1_ and T_2_ estimation. The diagonal entries of Figure 8A,C highlight the variability of repeat measures on the same vendor from standard RF conditions. As the voxel‐wise distributions are centered around zero for both T_1_ and T_2_, one can conclude that with the same vendor and with the same protocol, good reproducibility can be achieved. We find it important to highlight this result, which agrees with previous literature,^6^ in which each individual study reports a very tight range of normative tissue values. This is because typically the same qMRI protocol is measured at each site and hence good reproducibility is expected. Other issues arise when cross‐vendor comparison is sought, as highlighted in the off‐diagonal entries of Figure 8A,B. Nonzero centered variability distributions are observed, indicating systematic differences among different vendors even using the same acquisition and fitting strategy. Once again, this is in agreement with the recent review from Bojorquez et al,^6^ as different vendors will have different FAs, TRs, and pulse choices that will induce apparent T_1_ values, which are expected to deviate from one another, although each individual study is highly reproducible. As expected, once controlled saturation is achieved (Figure 8B,D), off‐diagonal entries are qualitatively indistinguishable from diagonal ones, as well as zero‐centered, demonstrating that cross‐vendor variability has been decreased to become comparable to single‐vendor test/retest scans.

To finalize, we compared how the results hold for different volunteers. To summarize this comparison, we plotted for each volunteer the median values of T_1_ and T_2_ in WM from the scans of all 3 different and with different protocols (Figure 9). The spread in these values is much tighter when using CSMT RF pulses, and unlike when vendor native sequences are used, there is no clear distinction among vendors. This corroborates our initial hypothesis that CSMT conditions allow significant increase in cross‐vendor reproducibility.

In this work we did not consider the accuracy of different B_1_ mapping approaches and used the B_1_ mapping approach that was already available on each of the scanners. It is well‐known that correct B_1_ accuracy and high precision are crucial to correctly estimate T_1_.^29^, ^30^ In addition, it has been recently suggested that some B_1_ map methodologies might be affected by MT effects.^31^ Further work might focus on establishing the reproducibility of different B_1_ mapping techniques to further reduce the cross‐vendor variability.

## 5 CONCLUSIONS

Magnetization transfer effects are a major contributor to intersite/intrasite variability of T_1_/T_2_ estimation across vendors. We demonstrate that harmonizing RF spoiling across all sites is a necessary but not sufficient condition to ensure reproducible results. With CSMT, MT effects are stabilized, allowing for significantly more reproducible measures across acquisition schemes and sites. Controlled saturation magnetization transfer paves the way for the use of T_1_ and T_2_ as a reliable source for clinical diagnosis across sites.

## Supporting information


**FIGURE S1** Comparison of T*_1_* (in milliseconds) compared across vendors using the data acquired from native RF spoiling and saturation conditions. All histograms were obtained from a single gray matter (GM) mask. Each color represents a different protocol, as per Table 1. Solid, dashed, and dotted lines correspond to vendor A, B, and C GM‐specific distributions, respectively
**FIGURE S2** Comparison of T*_2_* (in milliseconds) compared across vendors using the data acquired from native RF spoiling and saturation conditions. All histograms were obtained from a single GM mask. Each color represents a different protocol, as per Table 1. Solid, dashed, and dotted lines correspond to vendor A, B, and C GM‐specific distributions, respectively
**FIGURE S3** Cross‐vendor T*_1_* (in milliseconds) estimation comparison of the data acquired from each scanner’s native saturation conditions and harmonized RF spoiling of 50º. All histograms were obtained from a single GM mask. Each color represents different protocols, as per Table 1. Solid, dashed, and dotted lines correspond to vendor A, B, and C GM‐specific distributions, respectively
**FIGURE S4** Cross‐vendor T*_2_* (in milliseconds) estimation comparison of the data acquired from each scanner’s native saturation conditions and harmonized RF spoiling of 50º. All histograms were obtained from a single GM mask. Each color represents different protocols, as per Table 1. Solid, dashed, and dotted lines correspond to vendor A, B, and C GM‐specific distributions, respectively
**FIGURE S5** Cross‐vendor T*_1_* (in milliseconds) comparison of the data acquired from both harmonized RF spoiling and CSMT conditions. All histograms were obtained from a single GM mask. Each color represents different protocols, as per Table 1. Solid, dashed, and dotted lines correspond to A, B, and C GM‐specific distributions, respectively
**FIGURE S6** Cross‐vendor T*_2_* (in milliseconds) comparison of the data acquired from both harmonized RF spoiling and CSMT conditions. All histograms were obtained from a single GM mask. Each color represents different protocols, as per Table 1. Solid, dashed, and dotted lines correspond to A, B, and C GM‐specific distributions, respectively
