# Supplementary material for: Controlled saturation magnetization transfer for reproducible multivendor variable flip angle T1 and T2 mapping
Source: Magn Reson Med. 2019 Dec 17;84(1):221–36. doi: 10.1002/mrm.28109 (PMC7154666; doi:10.1002/mrm.28109)
Supplement: Supplementary file 1 — FIGURE S1 Comparison of T1 (in milliseconds) compared across vendors using the data acquired from native RF spoiling and saturation conditions. All histograms were obtained from a single gray matter (GM) mask. Each color represents a different protocol, as per Table 1. Solid, dashed, and dotted lines correspond to vendor A, B, and C GM‐specific distributions, respectively FIGURE S2 Comparison of T2 (in milliseconds) compared across vendors using the data acquired from native RF spoiling and saturation conditions. All histograms were obtained from a single GM mask. Each color represents a different protocol, as per Table 1. Solid, dashed, and dotted lines correspond to vendor A, B, and C GM‐specific distributions, respectively FIGURE S3 Cross‐vendor T1 (in milliseconds) estimation comparison of the data acquired from each scanner’s native saturation conditions and harmonized RF spoiling of 50º. All histograms were obtained from a single GM mask. Each color represents different protocols, as per Table 1. Solid, dashed, and dotted lines correspond to vendor A, B, and C GM‐specific distributions, respectively FIGURE S4 Cross‐vendor T2 (in milliseconds) estimation comparison of the data acquired from each scanner’s native saturation conditions and harmonized RF spoiling of 50º. All histograms were obtained from a single GM mask. Each color represents different protocols, as per Table 1. Solid, dashed, and dotted lines correspond to vendor A, B, and C GM‐specific distributions, respectively FIGURE S5 Cross‐vendor T1 (in milliseconds) comparison of the data acquired from both harmonized RF spoiling and CSMT conditions. All histograms were obtained from a single GM mask. Each color represents different protocols, as per Table 1. Solid, dashed, and dotted lines correspond to A, B, and C GM‐specific distributions, respectively FIGURE S6 Cross‐vendor T2 (in milliseconds) comparison of the data acquired from both harmonized RF spoiling and CSMT conditions. All histograms were o [file MRM-84-221-s001.pdf]

Supporting Information: Controlled Saturation Magnetization Transfer (CSMT) for Reproducible Multi-Vendor Variable Flip Angle T1 and T2 mapping

Supporting Information Figure S1

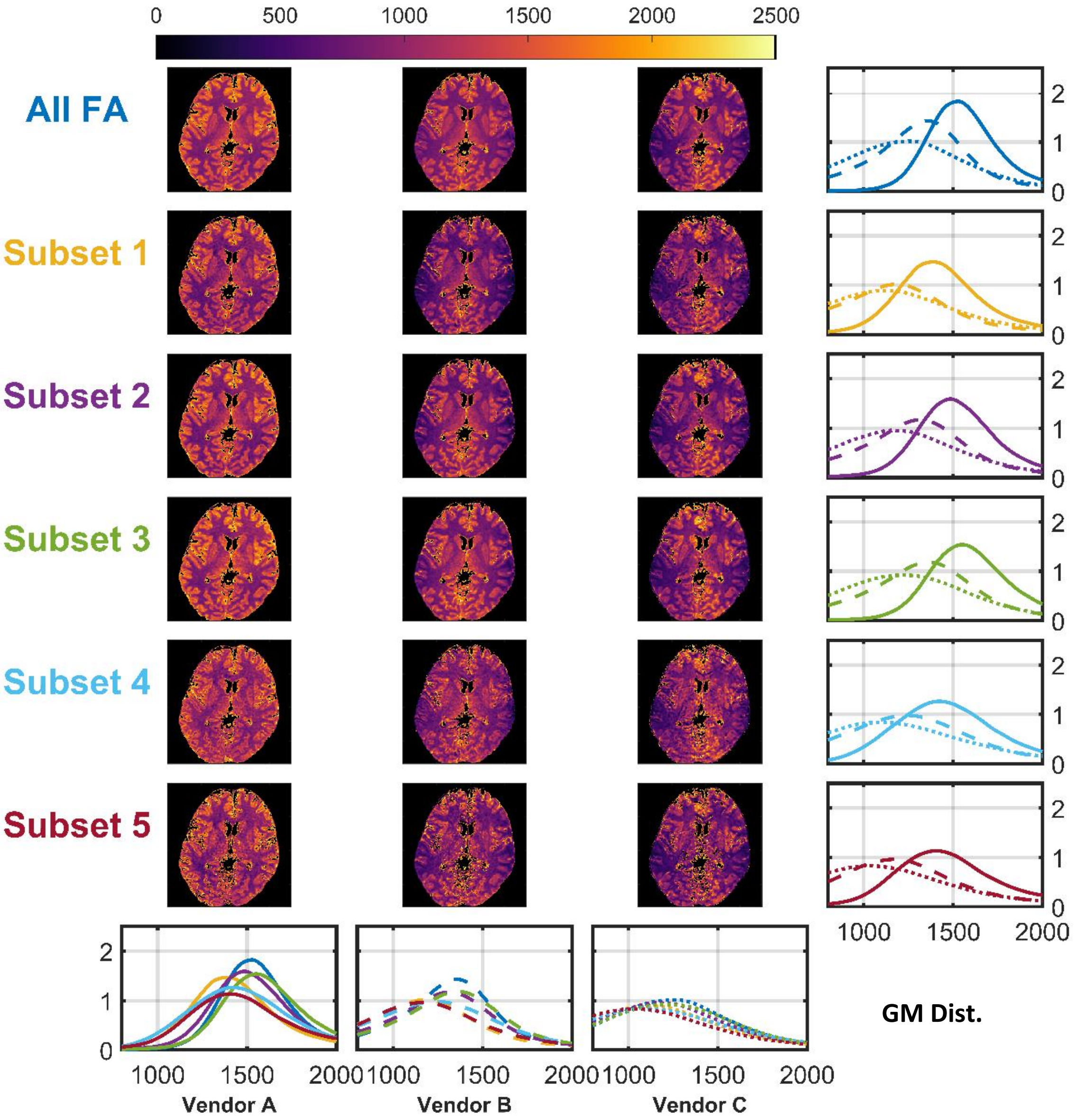

Supporting Information Figure S1 - Comparison of T1 (in ms) compared across vendors using data acquired with native RF spoiling and saturation conditions. All histograms are obtained from a single GM mask. Each colour represents a different protocol as per Table 1. Solid, dashed and dotted lines correspond respectively to vendor A, B and C GM specific distributions.

Supporting Information Figure S2

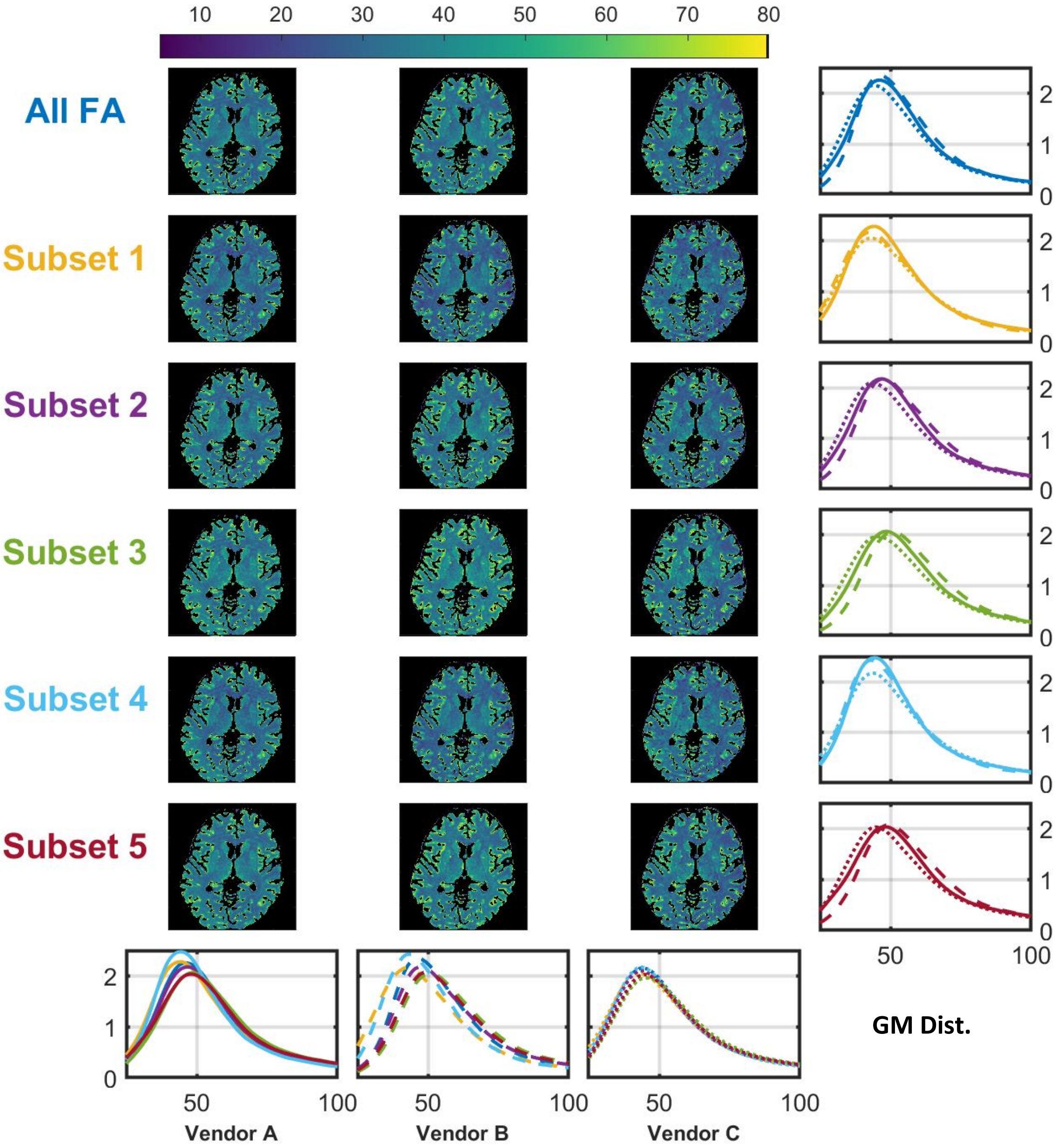

Supporting Information Figure S2 - Comparison of T2 (in ms) compared across vendors using data acquired with native RF spoiling and saturation conditions. All histograms are obtained from a single GM mask. Each colour represents a different protocol as per Table 1. Solid, dashed and dotted lines correspond respectively to vendor A, B and C GM specific distributions.

Supporting Information Figure S3

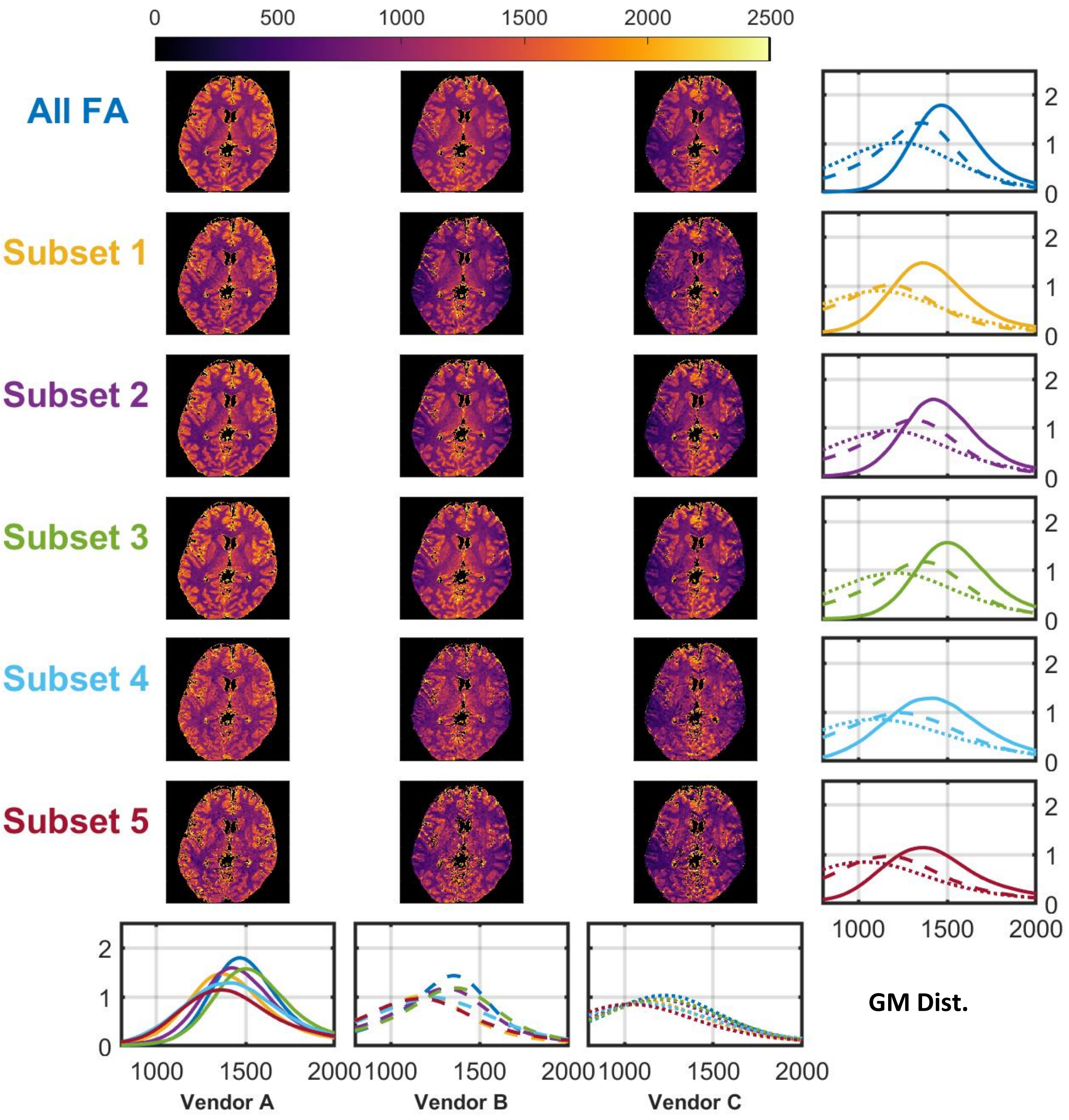

Supporting Information Figure S3 - Cross-vendor T1 (in ms) estimation comparison with data acquired with each scanner's native saturation conditions and harmonized RF spoiling of 50°; All histograms are obtained from a single GM mask. Each colour represents different protocols as per Table 1. Solid, dashed and dotted lines correspond respectively to vendor A, B and C GM specific distributions.

Supporting Information Figure S4

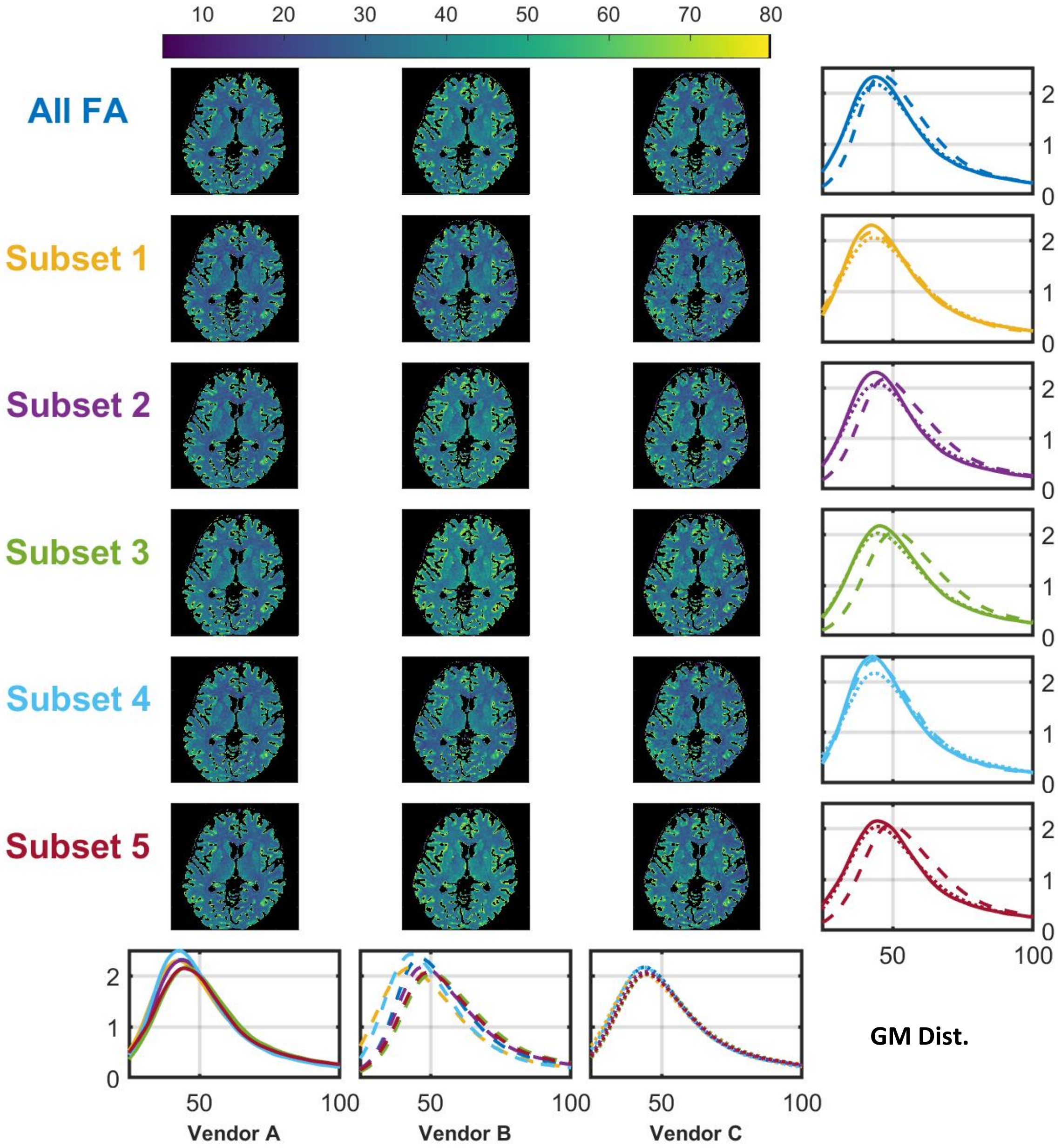

Supporting Information Figure S4 - Cross-vendor T2 (in ms) estimation comparison with data acquired with each scanner's native saturation conditions and harmonized RF spoiling of 50°; All histograms are obtained from a single GM mask. Each colour represents different protocols as per Table 1. Solid, dashed and dotted lines correspond respectively to vendor A, B and C GM specific distributions.

Supporting Information Figure S5

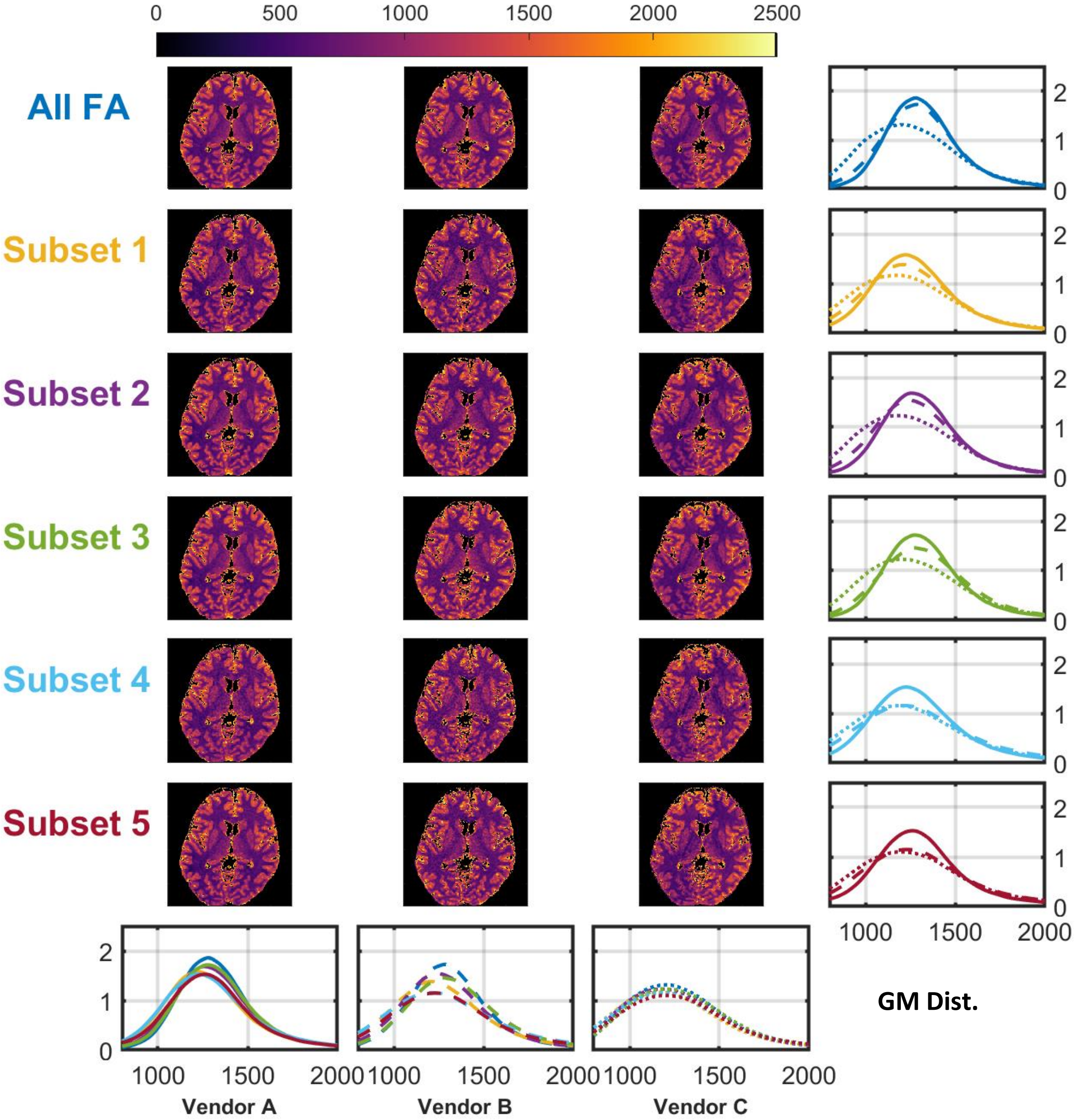

Supporting Information Figure S5 - Cross-vendor T1 (in ms) comparison with data acquired with both harmonized RF spoiling and CSMT conditions; All histograms were obtained from a single GM mask. Each colour represents different protocols as per Table 1. Solid, dashed and dotted lines correspond respectively to A, B and C GM-specific distributions

Supporting Information Figure S6

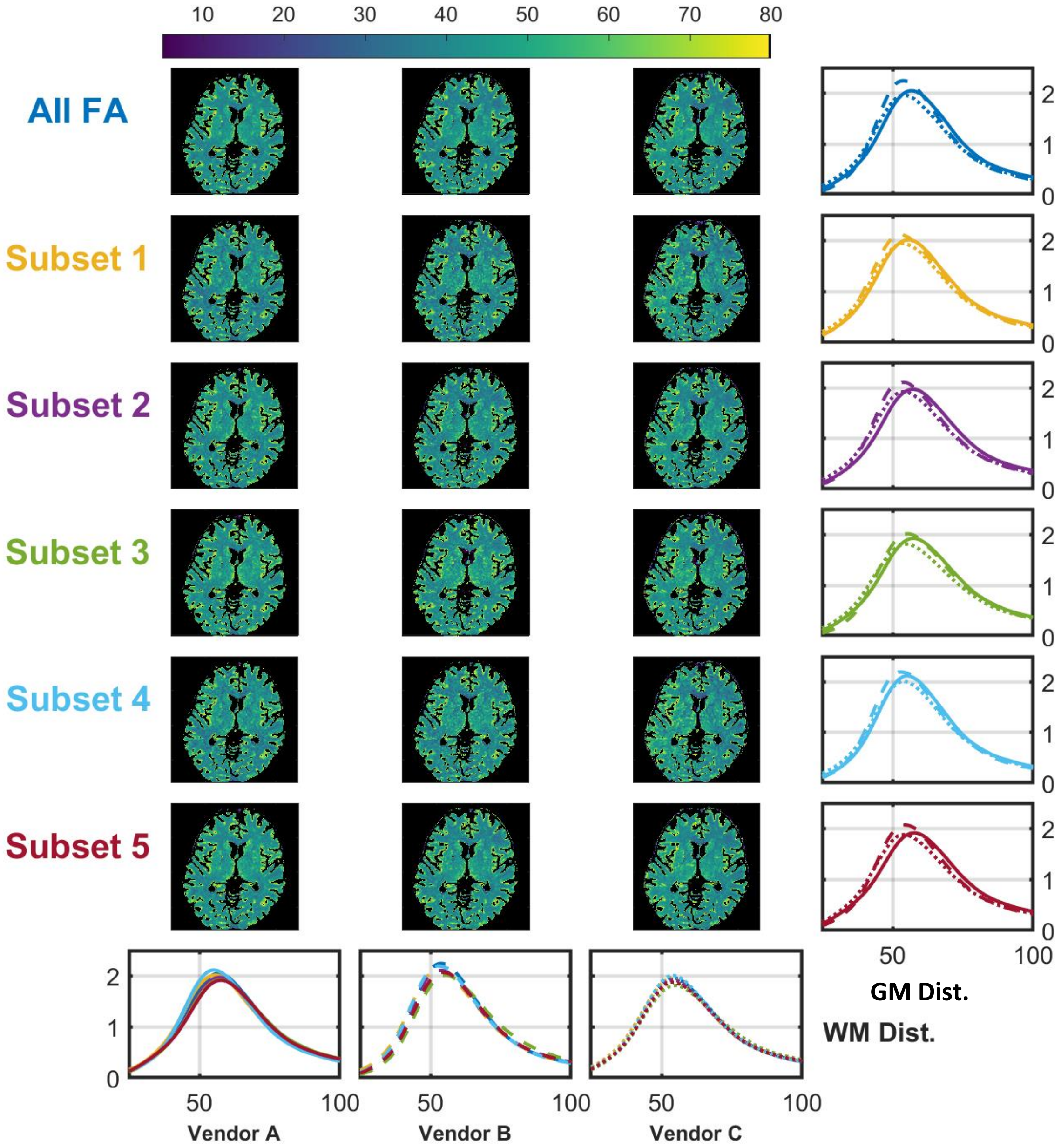

Supporting Information Figure S6 - Cross-vendor T2 (in ms) comparison with data acquired with both harmonized RF spoiling and CSMT conditions; All histograms were obtained from a single GM mask. Each colour represents different protocols as per Table 1. Solid, dashed and dotted lines correspond respectively to A, B and C GM-specific distributions
